# Strain-Divergent m6A Landscapes Modulate Nipah Virus Replication and METTL3 Inhibition Attenuates Virulence

**DOI:** 10.3390/v17060831

**Published:** 2025-06-09

**Authors:** Ting Luo, Zhen Chen, Fang Zhang, Haibin Liu, Fang Huang, Xueyan Zhang, Jiangpeng Feng, Shuang Ding, Lishi Liu, Wuxiang Guan, Aiping Zeng, Haojie Hao

**Affiliations:** 1 Center for Emerging Infectious Diseases, Wuhan Institute of Virology, Chinese Academy of Sciences, Wuhan 430207, China; 2 University of Chinese Academy of Sciences, Beijing 100049, China; 3 Hubei JiangXia Laboratory, Wuhan 430200, China; 4 Department of Ophthalmology, Union Hospital, Tongji Medical College, Huazhong University of Science and Technology, Wuhan 430022, China

**Keywords:** Nipah virus (NiV), N6-methyladenosine (m6A), virus pathogenicity, antiviral strategy, METTL3 inhibitor

## Abstract

Nipah virus (NiV), a highly lethal zoonotic paramyxovirus, displays strain-specific pathogenicity, yet the molecular basis for this divergence remains elusive. Here, we identify N6-methyladenosine (m6A) modification as a pivotal regulator of NiV replication. Higher m6A methylation levels on viral genomic RNA and mRNAs are associated with the increased virulence observed in the NiV-Malaysia (NiV-M) strain compared to NiV-Bangladesh (NiV-B). Underlying this phenomenon, NiV infection orchestrates a reprogramming of the host m6A machinery by downregulating the methyltransferase METTL3 and the demethylase ALKBH5, while concurrently upregulating m6A reader proteins YTHDF1-3. Both METTL3 and ALKBH5 bind directly to NiV RNA, with METTL3 installing m6A to promote viral replication and ALKBH5 removing them to inhibit it. Strikingly, pharmacological inhibition of m6A modification markedly attenuates NiV replication in vitro and in vivo, underscoring the therapeutic potential of targeting the m6A pathway. Our study establishes m6A as a key determinant of NiV pathogenicity and provides a paradigm for host-directed antiviral strategies against high-risk RNA viruses.

## 1. Introduction

Nipah virus (NiV), a member of the Henipavirus genus (Paramyxoviridae family), is a biosafety level 4 (BSL-4) pathogen with a high mortality rate (40–75%) and lack of approved antiviral therapies or vaccines [1]. Since it was identified during the 1998–1999 Malaysian outbreak linked to pig farms [2], NiV has caused recurrent epidemics in South and Southeast Asia, with over 700 documented human infections and over 400 deaths as of 2023. The virus is transmitted to humans via direct contact with infected bats, consumption of contaminated date palm sap, or through intermediate hosts such as pigs [3,4,5]. Human-to-human transmission occurs predominantly in NiV-Bangladesh (NiV-B) outbreaks, contributing to its higher epidemic potential. Understanding NiV replication mechanisms and pathogenic drivers is critical for developing targeted interventions to curb its zoonotic spread and mitigate public health crisis.

The NiV genome comprises a non-segmented, single-stranded negative-sense RNA (~18.2 kb) encoding six structural proteins (nucleoprotein N, phosphoprotein P, matrix protein M, fusion protein F, attachment glycoprotein G, large polymerase L) and three accessory proteins (V, W, C) that suppress host antiviral responses [6,7,8,9,10,11,12,13]. Viral mRNA transcription and replication are mediated by the RNA-dependent RNA polymerase (L protein), while the F and G glycoproteins orchestrate membrane fusion and cell entry, respectively. Based on geographic origin and genetic divergence, NiV is classified into two strains: NiV-Malaysia (NiV-M) and NiV-B [14,15,16]. NiV-M primarily spreads to humans through direct contact with infected animals, particularly pigs, during zoonotic spillover events. While rare instances of asymptomatic seroconversion or delayed encephalitis in close contacts have been reported for NiV-M, no sustained human-to-human transmission chains have been documented. In contrast, NiV-B is associated with frequent human-to-human transmission via respiratory secretions or bodily fluids, contributing to its higher epidemic potential [17,18,19]. Deciphering the molecular basis of these strain-specific pathogenic differences is essential for understanding their differential virulence and guiding the development of targeted antiviral strategies. 

N6-methyladenosine (m6A) is the most widely prevalent modification found in eukaryotic messenger RNAs (mRNAs) and non-coding RNAs [20,21,22]. It is installed, removed, and interpreted by a set of evolutionarily conserved proteins: the “writers” (methyltransferases such as METTL3/METTL14), “erasers” (demethylases including FTO and ALKBH5), and “readers” (m6A-binding proteins such as YTHDF1-3 and YTHDC1/C2) [23,24,25,26,27,28,29,30,31,32,33,34,35,36,37,38]. These factors dynamically regulate RNA metabolism, including splicing, stability, translation, and localization [32,35,39,40,41,42,43]. In recent years, mounting evidence has revealed that a wide array of viruses exploit this m6A machinery to manipulate host cell biology and fine-tune their own replication, immune evasion, and pathogenicity [44]. For instance, Human immunodeficiency virus type 1 (HIV-1) leverages METTL3/METTL14-mediated m6A deposition on the Rev response element (RRE) to facilitate Rev binding and nuclear export of viral RNA, while also utilizing m6A to enhance infectivity and evade RIG-I sensing by YTHDF recruitment [45,46,47,48,49]. In Severe acute respiratory syndrome coronavirus 2 (SARS-CoV-2), METTL3-driven m6A modification stabilizes viral RNAs and aids immune evasion by masking viral RNAs from RIG-I recognition, with RNA-dependent RNA polymerase (RdRp) directly interacting with METTL3 to boost methylation activity [50,51,52]. Similarly, Influenza A virus (IAV) benefits from METTL3-mediated m6A modification to enhance viral protein expression, splicing regulation, RdRp function, and pathogenicity [53,54,55]. Among Flaviviruses, such as Zika virus (ZIKV) and Hepatitis C virus (HCV), m6A exerts both pro- and anti-viral effects depending on the localization of m6A peaks, with METTL3/METTL14 and YTHDF2 facilitating immune escape or modulating RNA stability and particle release [56,57,58,59,60,61]. Additionally, Enterovirus 71 (EV71) employs m6A to promote replication and immune evasion [62,63], while pneumoviruses like Respiratory syncytial virus (RSV) and Human metapneumovirus (hMPV) exploit m6A-modified transcripts to circumvent RIG-I-mediated antiviral responses [64,65]. Furthermore, other negative-sense RNA viruses such as Vesicular stomatitis virus (VSV), Ebola virus (EBOV), and Crimean–Congo hemorrhagic fever virus (CCHFV) also hijack the host m6A machinery to promote replication and evade innate immunity. VSV infection induces cytoplasmic relocalization of METTL3, which methylates viral RNAs to reduce dsRNA accumulation and suppress RIG-I/MDA5 signaling [66]. In EBOV and CCHFV, METTL3 is recruited to viral inclusion bodies and interacts with viral proteins to support RNA synthesis and replication, highlighting a conserved role of m6A in facilitating viral life cycles [67]. These findings underscore m6A as a critical modulator of virus–host interactions and a potential target for antiviral therapies.

NiV poses significant challenges in therapeutic development due to its high pathogenicity and the necessity for Biosafety Level-4 (BSL-4) containment, which limits extensive research. Currently, there are no clinically approved treatments for NiV infection, underscoring the urgent need for novel therapeutic strategies. Existing antiviral drug development often focuses on inhibiting viral RdRp, with agents such as remdesivir [68,69] and favipiravir [70] being prominent examples. However, these drugs have limitations, including variable efficacy and the potential for resistance development. Targeting m6A RNA modification pathways offers a promising alternative for antiviral therapy, given their crucial roles in viral replication and host immune responses. For instance, the use of 3-deazaadenosine (DAA), an m6A methylation inhibitor, resulted in over a 1000-fold reduction in Herpes simplex virus type 1 (HSV-1) reproduction [71].

This study aimed to elucidate the role of m6A dynamics in shaping NiV replication efficiency and inter-strain pathogenicity. The m6A landscapes of NiV-M and -B strains were mapped, host m6A machinery–virus interplay was dissected, and antiviral potency of methylation inhibitors was validated. These findings advance the understanding of NiV–host interplay and highlight m6A-centric approaches as a novel therapeutic paradigm against highly pathogenic henipaviruses.

## 2. Materials and Methods

### 2.1. Cell Culture

Vero (CCL-81) and HEK293T (CRL-11268) cells were obtained from the American Type Culture Collection (ATCC; Manassas, VA, USA). Cells were cultured in Dulbecco’s modified Eagle’s medium (DMEM; Gibco, Gaithersburg, MD, USA) supplemented with 10% fetal bovine serum (FBS; Gibco) at 37 °C and 5% CO_2_.

### 2.2. Viruses

NiV strains (NiV-M and NiV-B) were obtained from the Microorganisms & Viruses Culture Collection Center at the Wuhan Institute of Virology (WIV), Chinese Academy of Sciences (CAS). The viruses were propagated in Vero cells and titrated using the 50% tissue culture infectious dose (TCID50) assay, following the Reed–Muench method. All infectious work involving NiV was performed in the BSL-4 facility at the Wuhan National Biosafety Laboratory (Approval number: NBL4C-24002).

### 2.3. Plasmid Constructs

METTL3, ALKBH5, and FTO eukaryotic expression plasmids, pFlag-METTL3, pFlag–ALKBH5, and pFlag–FTO, were generated by cloning the coding sequence (CDS) of METTL3, ALKBH5, or FTO from Chlorocebus aethiops into the pXJ40-Flag vector (Sigma-Aldrich, St. Louis, MO, USA), respectively. pMyc-L, derived from the NiV-M strain, was cloned into the pCAGGS vector (MIAOLING BIOLOGY). pFlag-N, pFlag-P, pFlag-G, pFlag-F, and pFlag-M derived from the NiV-M strain were cloned into the pCMV-3×Flag vector, respectively.

### 2.4. Hamster

Six-week-old hamsters were randomly assigned to experimental groups for NiV infection. All animal procedures adhered to the institutional guidelines and were approved by the Institutional Animal Care and Use Committee of WIV, CAS (Approval no. WIVAF32202401). The humanitarian endpoint of the hamster study is a weight loss of more than 25%.

### 2.5. Antibodies and Reagents

The primary antibodies used in this study included mouse monoclonal antibodies against GAPDH (60004-1-lg, Proteintech, Rosemont, IL, USA), ALKBH5 (67811-1-lg, Proteintech), Flag (F1804 MG, Sigma-Aldrich), NiV F (MAB21940, Abnova, Taipei, China), and m6A (ab151230, abcam, Cambridge, UK); rabbit polyclonal antibody against NiV G (CABT-L11VR, CD Creative Diagnostics, Shirley, NY, USA), NiV N (NIV21-A, Alpha Diagnostic, San Marcos, TX, USA), METTL3 (15073-1-AP, Proteintech), METTL14 (26158-1-AP, Proteintech), YTHDF1 (17479-1-AP, Proteintech), YTHDF2 (24744-1-AP, Proteintech), YTHDF3 (25537-1-AP, Proteintech), FTO (27226-1-AP, Proteintech); control antibodies including mouse IgG (B900620, Proteintech) and rabbit IgG (30000-0-AP, Proteintech); and a custom mouse polyclonal antibody against Nipah M protein generated by AtaGenix Laboratories Co., Ltd. (Wuhan, China). Secondary antibodies included goat anti-mouse IgG and goat anti-rabbit IgG (AntiGene Biotech GmbH, Stuttgart, Germany).

### 2.6. Ultra-High-Performance Liquid Chromatography–Tandem Mass Spectrometry (UPLC-MS/MS)

UPLC-MS/MS was performed by Wuhan Metware Biotechnology Co., Ltd. (Wuhan, China). NiV-M and NiV-B supernatants were concentrated and purified using dynabeads according to the instructions from the manufacturer (Invitrogen, Carlsbad, CA, USA). NiV RNAs were extracted employing the RNeasy mini kit (Qiagen, Valencia, CA, USA) and enzymatically digested into nucleosides with S1 nuclease (Takara, Shiga, Japan), alkaline phosphatase (Takara), and phosphodiesterase I (Sigma-Aldrich). The nucleosides were then extracted with chloroform and analyzed by a UPLC-ESI-MS/MS system (UPLC: ExionLC AD; MS: Applied Biosystems 6500 Triple Quadrupole). RNA modifications were detected using MetWare (http://www.metware.cn/, accessed on 6 January 2022) on the AB Sciex QTRAP 6500 LC-MS/MS platform.

### 2.7. Quantitative Reverse-Transcription PCR (qRT-PCR)

The RNAs were extracted using TRIzol reagent (Invitrogen, Cat. No. 15596026). Reverse transcription was conducted with ABScript III RT Master Mix for qPCR with gDNA Remover (ABclonal, Wuhan, China). qRT-PCR was performed using Hieff^®^ qPCR SYBR GreenMaster Mix (No Rox) (Yeasen, Shanghai, China) on a CFX Connect Real-Time system (Bio-Rad). Relative gene expression levels were determined by normalizing quantification Cycle (Cq) values to those of *GAPDH*, yielding 2^−△△Cq^. For formaldehyde RIP-qPCR and MeRIP-qPCR, relative enrichment was normalized to input levels. The primers used for gene expression analysis included NiV N (forward: 5′-CCATTATTGTGGAGCTTTG-3′, reverse: 5′-GAACTTAGTCCCAGTCTATTTGC-3′), NiV P (forward: 5′-GGACGTACAATCGAAGGCCA-3′, reverse: 5′-TAGCCTCTTCTGGGGTGGTT-3′), NiV F (forward: 5′- AACAACAGGCAGGGCAATCT-3′, reverse: 5′-AGACTGGAGGACCGATAGCA-3′), NiV G (forward: 5′- AAGACCCGGGCAATCACAAT-3′, reverse: 5′-TTCTGCGGTCTGATTGCTGT-3′), NiV L (forward: 5′- CGAAGTCAACCGAGCCAAGA-3′, reverse: 5′-GTCCAGCTTGCTTCGTCTCT-3′), *METTL3* (forward: 5′-CACTGATGCTGTGTCCATCT-3′, reverse: 5′-CTTGTAGGAGACCTCGCTTTAC-3′), *ALKBH5* (forward: 5′-GCTGTATCAGGCTGGGTTTAT-3′, reverse: 5′-GTACAGACATCAGTGGAGGAAAG-3′), *FTO* (forward: 5′-AATAGCCGCTGCTTGTGAGA-3′, reverse: 5′-TCCACTTCATCTTGTCCATCG-3′), *GAPDH* (forward: 5′-GAAGGTGAAGGTCGGAGT C-3′, reverse: 5′-GAAGATGGTGATGGGATTTC-3′), and *β-actin* for hamster (forward: 5′-GGCCAGGTCATCACCATT-3′, reverse: 5′-GAGTTGAATGTAGTTTCGTGGATG-3′).

### 2.8. m6A-Methylated RNA Immunoprecipitation (MeRIP) and qRT-PCR

Total RNA was extracted from NiV-M- or NiV-B-infected Vero cells (MOI = 0.01) using TRIzol reagent. Of the extracted RNA, 100 µg was used for the assay, with 1 µg reserved as input and the remainder incubated with an anti-m6A antibody or control IgG antibody in 600 µL of IP buffer (150 mM NaCl, 10 mM Tris-HCl, pH 7.4, 0.1% NP-40) overnight at 4 °C. The mixture was then incubated with 20 µL Dynabeads^®^ Protein A and 20 µL Protein G (Invitrogen) for 2 h at 4 °C. After incubation, the beads were washed six times with 800 µL of IP buffer, and RNA was extracted using TRIzol reagent. The levels of NiV RNA in the input and immunoprecipitated samples were quantified by qRT-PCR.

### 2.9. Western Blot Analysis

Cells were seeded at 80% confluence 1 day prior to infection with NiV or transfection with plasmids. At the indicated time, cells were harvested and lysed. The lysates were centrifuged, quantified, and denatured by boiling in a loading buffer for 10 min. Samples were then separated by SDS-polyacrylamide gel electrophoresis. Western blot analysis was performed, adhering to a standard protocol [72].

### 2.10. Formaldehyde-Crosslinked RNA-Immunoprecipitation and qRT-PCR

The cells were first transfected with plasmids pFlag-METTL3 and -ALKBH5, followed by transfection of NiV-P, -G, and -L RNAs after 36 h. They were then crosslinked with 1% methanol-free formaldehyde in PBS at 37 °C for 10 min. Crosslinking was stopped by adding 2.5 M glycine to a final concentration of 0.125 M. After three ice-cold PBS washes, cells were scraped and centrifuged at 800× *g* for 3 min at 4 °C. Pellets were resuspended in 600 μL RIP buffer (150 mM KCl, 25 mM Tris-HCl (pH 7.4), 5 mM EDTA, 0.5 mM DTT, 0.5% NP-40, 100 U/mL RNase inhibitor, 100 µM PMSF, and 1 μg/mL proteinase inhibitors) and incubated on ice for 30 min. Lysates were centrifuged at 13,000× *g* at 4° for 10 min, and supernatants containing protein–RNA complexes were subjected to IP overnight with anti-METTL3, anti-ALKBH5, mouse IgG control (control), and rabbit IgG (control), respectively. The next day, 30 μL of Dynabeads^®^ Protein A and 30 µL of Protein G were added to each sample and incubated for 2 h at 4 °C. Beads were washed three times with washing buffer (300 mM KCl, 25 mM Tris-HCl (pH 7.4), 5 mM EDTA, 0.5 mM DTT, 0.5% NP-40, 100 U/mL RNase inhibitor, 100 µM PMSF, and 1 μg/mL proteinase inhibitors), followed by three additional RIP buffer washes. RNA was extracted using TRIzol and quantified by qRT-PCR.

### 2.11. shRNA and siRNA-Mediated Gene Silencing

The shRNA sequences targeting METTL3 included Vero cell (shMETTL3-98: 5′-GCCAAGGAACAATCCATTGTT-3′, shMETTL3-96: 5′-GCTGCACTTCAGACGAATTAT-3′). These sequences were cloned into the pLKO.1-TRC vector (Addgene plasmid 10878) and co-transfected with psPAX and pMD2G into HEK293T cells to package lentiviruses. Stable knockdown cell lines were generated through lentiviral infection, followed by puromycin selection at 10 μg/mL for Vero cells. The siRNA target sequence were siALKBH5-1: 5′-GCUUCAGCUCUGAGAACUA-3′, siALKBH5-2: 5′-GGAUAUGCUGCUGAUGAAA-3′, siFTO-1: 5′-GGACCUGGUUAGGAUCCAA-3′, siFTO-2: 5′-CAAGGCAAUCGAUACAGAA-3′.

### 2.12. Nanopore Direct RNA Sequencing (DRS)

Nanopore DRS was conducted as previously described [50]. Briefly, RNA was extracted from 1 mg of NiV-M or -B-infected Vero cells using an Oligo (dT) purification kit (Thermo Scientific, Waltham, MA, USA). Libraries were prepared following the Oxford Nanopore DRS protocol (SQKRNA002) and sequenced on a MinION device (Oxford Nanopore Technologies, Oxford, UK). The NiVM (AF212302.2) and B (AY988601.1) served as the viral reference genome, and DRS data were analyzed by BENAGEN (Nanopore Company, Oxford, UK). A threshold with a Q value of 7 was set to obtain pass reads, and base-calling was conducted using guppy v3.4.5 (Oxford Nanopore Technologies). The multi_to_single_fast5 of ont_fast5_api (v3.1.6; https://github.com/nanoporetech/ont_fast5_api, accessed on 24 May 2022) was used to convert multi-fast5 reads into single reads, followed by MINES analysis [73]. Tombo (v1.5) was used to resquiggle the fast5 data with default parameters. The de novo noncanonical base detection mode (tombo detect_modifications de_novo–coverage-dampen-counts 0 0) was applied to identify modified bases at each position on the viral genome and assess methylation ratios and coverage for each base. m6A sites were identified using MINES (cDNA_MINES.py, default parameters), and motifs were visualized with ggseqlogo package (vggseqlogo_0.1) [74].

### 2.13. Infection of Hamster

Hamsters were inoculated intraperitoneally (i.p.) with 500 μL of DMEM containing 500LD_50_ of NiV per hamster. Morbidity was assessed by daily weight monitoring. For RNA quantification, hamsters were euthanized at 4 days post-infection, and lung and spleen tissues were collected.

### 2.14. Statistical Analysis

Statistical analyses were performed using GraphPad Prism (version 8.0). Normality was assessed with the Shapiro–Wilk test, and variance homogeneity with the F-test (two groups) or Brown–Forsythe test (multiple groups). For normally distributed data with equal variances, we used unpaired Student’s *t*-tests or one-way ANOVA as appropriate. If variances were unequal, the Welch’s correction was applied. Two-way ANOVA was used for analyses involving two independent variables. *p* < 0.05 was considered statistically significant. Specific tests are indicated in the figure legends.

## 3. Results

### 3.1. Positive Correlation Between m6A Modification Levels and Replication Capacity of NiV Strains

**Figure 1:**
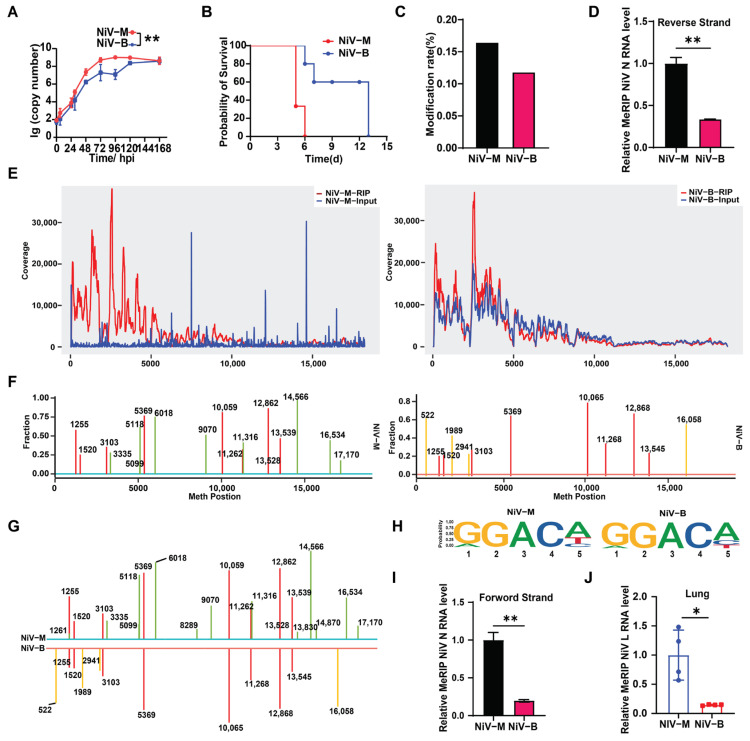
Positive correlation between m6A modification levels and replication capacity of NiV strains. (**A**) NiV RNA copy numbers in supernatants collected at various time points post-infection were quantified by absolute qPCR. Data are means G SEMs (*n* = 3). ** *p* < 0.01, two-way ANOVA with Holm–Sidak’s multiple comparisons test. (**B**) Analysis of published data comparing the impact of NiV-M and NiV-B strains on hamster survival under identical treatment conditions. (**C**) UHPLC-MS/MS analysis of NiV RNA. Viral RNA from supernatants of NiV-infected Vero cells was purified using Dynabeads and analyzed by UHPLC-MS/MS. The *y*-axis shows m6A as a percentage of total adenosines. (**D**) MeRIP-qRT-PCR analysis. RNA from NiV-infected Vero cells was immunoprecipitated with anti-m6A antibodies, followed by strand-specific qRT-PCR to detect the NiV negative-strand genome. Data are presented as means ± SEMs (*n* = 3). ** *p* < 0.01, unpaired Student’s *t*-tests. (**E**) MeRIP-Seq. Fragmented viral RNA from the supernatant of NiV-infected Vero cells was immunoprecipitated with an m6A-specific antibody and analyzed by next-generation sequencing. Methylation coverage of input and MeRIP-enriched NiV RNA is shown in blue and red, respectively. (**F**) Nanopore DRS analysis of NiV-M and NiV-B mRNAs from infected Vero cells. The *y*-axis shows m6A methylation probability at each A site; only sites with >95% probability are shown. Shared m6A sites between both strains are marked in red, while NiV-M- and NiV-B-specific sites are highlighted in green and yellow, respectively. (**G**,**H**) Bioinformatics analysis of m6A distribution and conserved motifs in NiV-M and NiV-B mRNAs. (**I**) MeRIP-qRT-PCR. RNA from NiV-infected Vero cells was immunoprecipitated with anti-m6A antibodies, followed by strand-specific qRT-PCR for NiV mRNA. Data are mean ± SEM (*n* = 3); ** *p* < 0.01, unpaired Student’s *t*-test. (**J**) MeRIP-qRT-PCR analysis of m6A-modified viral RNA in lung tissues of NiV-infected hamsters. Total RNA was extracted from lung tissue, immunoprecipitated using anti-m6A antibodies, and subjected to qRT-PCR targeting NiV mRNA. Data are presented as mean ± SEM (*n* = 4); * *p* < 0.05, unpaired Student’s *t*-test with Welch’s correction.

NiV exists as two primary strains (NiV-M and NiV-B) with differential outbreak patterns. To determine whether the two strains differ in replication capacity, viral growth curves were generated by quantifying RNA levels in NiV-infected Vero cell supernatants over time. NiV-M consistently exhibited higher RNA copy numbers than NiV-B at all assessed time points (Figure 1A). This replication advantage was further corroborated by analysis of prior literature data [75], demonstrating that in a hamster infection model, NiV-M-infected animals exhibited prolonged survival compared to NiV-B-infected counterparts under standardized challenge conditions (Figure 1B). Together, NiV-M exhibited superior replication capacity in both cellular and animal models.

Emerging evidence links m6A modifications to the regulation of viral replication and pathogenicity across diverse viral species [44,76]. Replication differences between NiV strains in relation to m6A were investigated by purifying viral particles from the supernatants of NiV-M- and NiV-B-infected cells using charge-specific magnetic beads that efficiently bind enveloped viruses, followed by genomic RNA extraction. UPLC-MS/MS analysis revealed the presence of m6A modifications in both strains, with NiV-M exhibiting a higher modification rate than that of NiV-B (Figure 1C). The m6A modifications on the RNAs of both NiV strains were further validated by using MeRIP-qPCR (Figure 1D). To map the m6A distribution, genome-wide analysis of supernatant-derived viral RNA via MeRIP-seq was performed. The m6A peaks on the genomic RNA of both NiV-M and NiV-B were predominantly distributed at the 3′ end (Figure 1E). Compared to the Input, NiV-M exhibited higher peaks, indicating a greater level of m6A modification in the NiV-M genome.

NiV contains a negative-sense genomic RNA and generates polyadenylated positive-sense mRNAs during replication [6,7,8,9,10]. The m6A modification landscape of NiV mRNAs was systematically profiled using Nanopore direct RNA sequencing, which exploits the polyadenylated structure of viral transcripts to specifically enrich positive-sense RNAs while excluding genomic RNA contamination. A broad distribution of m6A modifications on NiV mRNA (Figure 1F) was observed, with conserved modification motifs identified in both NiV-M and NiV-B (Figure 1H). Strikingly, NiV-M exhibited a greater number of m6A-modified sites compared to NiV-B, although multiple shared m6A sites were identified between both strains (Figure 1G), which further validated by MeRIP-qPCR (Figure 1I). To validate whether this strain-specific m6A pattern extends to in vivo contexts, we analyzed lung tissues from NiV-infected hamsters. Consistent with in vitro results, NiV-M viral RNA extracted from lung tissue exhibited higher m6A levels than that of NiV-B (Figure 1J). Collectively, NiV-M possesses enhanced replication capacity relative to NiV-B, with m6A modification levels on both viral genomic RNA and mRNA positively correlating with replication efficiency. This underscores the potential regulatory role of m6A in NiV replication dynamics.

The mechanisms underlying the m6A differences between NiV-M and NiV-B were investigated. As m6A methylation requires the presence of AAC or GAC conserved sequences, the numbers of AAC and GAC motifs in the genomes of both NiV-M and -B were initially analyzed to determine whether the differences in m6A modification could be attributed to variations in the number of potential m6A methylation sites. Intriguingly, NiV mRNA contained more AAC and GAC motifs than the viral genome RNA, but there was no significant difference between the two strains. This suggests that the variation in m6A levels is not due to differences in conserved sequences (Table 1).

### 3.2. NiV Modulates the Host m6A Modification System

Many viruses are known to alter the expression of m6A-related proteins during infection [62]. We hypothesize that the variation in m6A modification levels between the NiV-M and NiV-B strains is due to their distinct impacts on host m6A methyltransferases and demethylases. Transcriptomic analysis revealed that the expression changes of m6A-associated proteins following NiV infection were overall modest, and the majority did not reach statistical significance (Table 2). Nevertheless, NiV-M infection tended to retain higher residual METTL3 levels and exhibited a greater degree of ALKBH5 suppression compared to NiV-B (Table 2). Concurrently, a slight increase in YTHDF2/3 expression was observed. Although most of these differences did not reach statistical significance, they are consistent with the observed higher m6A modification levels in NiV-M (Figure 1C–I).

**Figure 2:**
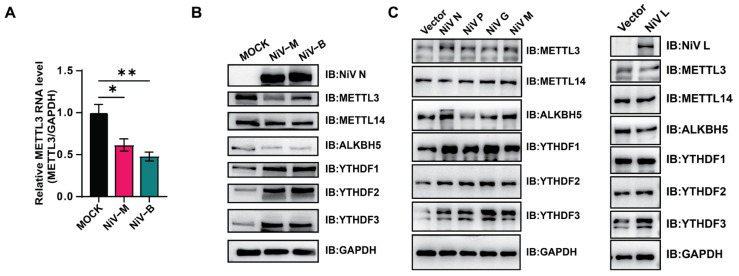
NiV modulates the host m6A modification system. (**A**) qRT-PCR analysis of METTL3 RNA levels following NiV infection. Data are mean ± SEM (*n* = 3); ** *p* < 0.01, * *p* < 0.05, one-way ANOVA with Dunnett’s multiple comparisons test. (**B**) Western blot analysis of m6A-related protein expression in mock- or NiV-infected cells. (**C**) Western blot of m6A-related proteins after overexpression of NiV N, P, G, or L.

We further validated the transcriptional downregulation of METTL3 by qRT-PCR (Figure 2A), where a more pronounced decrease was observed in NiV-B-infected cells. Western blot analysis also mirrored these transcriptional patterns, demonstrating reduced expression of METTL3/14 and ALKBH5, along with upregulation of m6A reader proteins YTHDF1-3 following NiV infection (Figure 2B). Together, these data suggest that subtle, strain-dependent modulation of the host m6A machinery may underlie the differential m6A enrichment observed between NiV-M and NiV-B.

Viral proteins frequently modulate host protein networks. NiV’s impact on the m6A modification system was examined through expression of its structural proteins. In addition to the L protein, P, G, M, and N proteins independently induced upregulation of m6A reader proteins YTHDF1-3, while maintaining stable expression levels of methyltransferases METTL3/14 and demethylase ALKBH5 (Figure 2C). These observations suggest that multiple NiV proteins can influence the expression of YTHDF proteins, while the regulation of m6A methyltransferases and demethylases likely involves a more complex mechanism beyond the action of a single viral protein.

### 3.3. m6A Promotes NiV Replication

**Figure 3:**
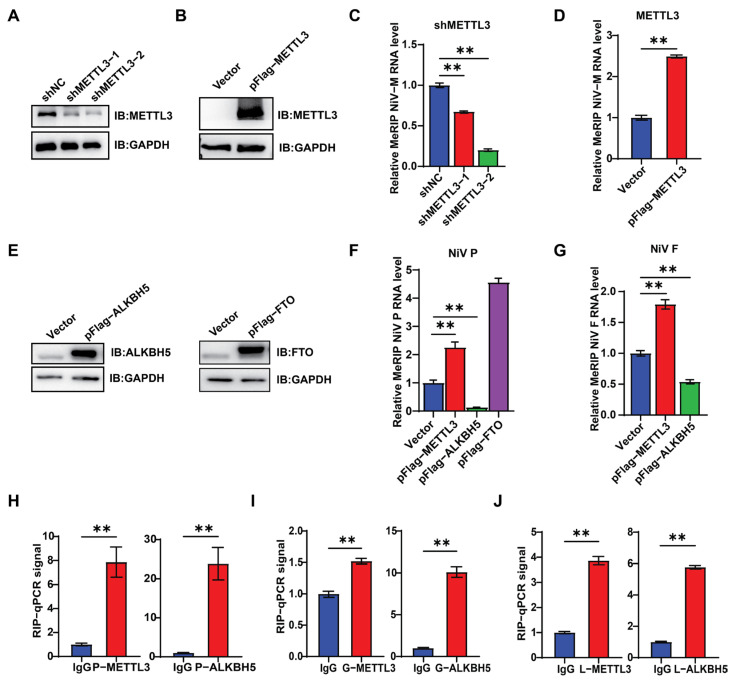
METTL3 and ALKBH5 mediate the methylation and demethylation of NiV RNA m6A. (**A**,**B**,**E**) Western blot detection of the expression of the corresponding proteins in Vero cells treated with shMETTL3, pFlag-METTL3, pFlag-FTO, and pFlag-ALKBH5. (**C**,**D**) MeRIP-qRT-PCR detection of the m6A modification levels of viral RNA in NiV-infected Vero cells with METTL3 knockdown (**C**) and overexpressed (**D**). (**C**) Data are mean ± SEM (*n* = 3); ** *p* < 0.01, one-way ANOVA with Dunnett’s multiple comparisons test. (**D**) Data are mean ± SEM (*n* = 3); ** *p* < 0.01, unpaired Student’s *t*-test. (**F**,**G**) MeRIP-qRT-PCR detection of m6A modification levels of NiV P or NiV F RNA in pNiV-P or NiV-F-transfected Vero cells with overexpression of METTL3, ALKBH5, or FTO. Data are mean ± SEM (*n* = 3); ** *p* < 0.01, one-way ANOVA with Dunnett’s multiple comparisons test. (**H**–**J**) Formaldehyde-RIP-qPCR. Vero cells transfected with NiV P, G, or L were crosslinked with formaldehyde, lysed, and subjected to immunoprecipitation (IP) using anti-METTL3 or ALKBH5 antibodies, or IgG as a control, followed by quantification via qRT-PCR. Unpaired Student’s *t*-test was performed, and data are presented as the means ± SEM (*n* = 3). ** *p* < 0.01.

NiV does not encode m6A-related proteins, indicating that NiV RNA methylation might depend on the host m6A modification system. To this end, Vero cells were subjected to METTL3 knockdown using shRNA or overexpression using pFlag-METTL3 (Figure 3A,B), followed by NiV infection and MeRIP-qPCR. m6A levels in viral RNA increased with METTL3 overexpression or decreased upon its knockdown (Figure 3C,D). pNiV-P and pMETTL3 were co-transfected into cells to further validate the effect of METTL3 on NiV mRNA. Consistent with observations in NiV-infection, METTL3 enhanced m6A modification of NiV P mRNA. Moreover, overexpression of the demethylases ALKBH5 and FTO revealed that ALKBH5, but not FTO, reduced m6A modification of P mRNA. This implies that NiV m6A modification is predominantly regulated by METTL3 and ALKBH5. A similar trend was observed when pNiV-F transfected (Figure 3E–G). Moreover, METTL3 and ALKBH5 interact with mRNAs of NiV P, NiV G, and NiV L, which was demonstrated by formaldehyde crosslinking RIP-qRT-PCR (Figure 3H–J), implying that METTL3 and ALKBH5 are the methyltransferase and demethylase for NiV RNAs, respectively.

**Figure 4:**
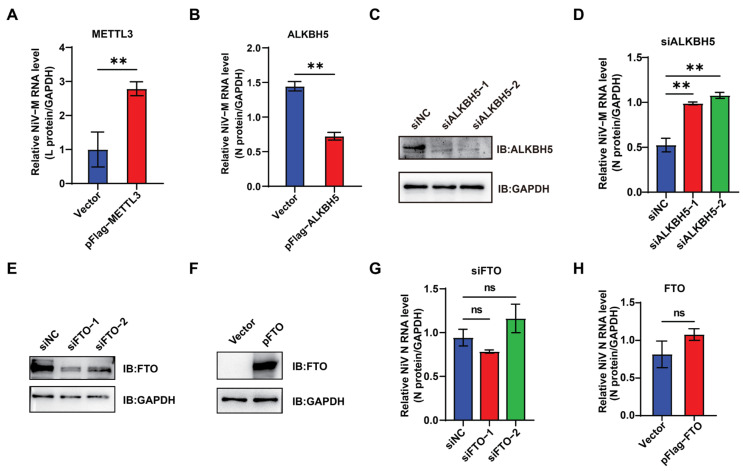
m6A promotes NiV replication. (**A**,**B**,**D**,**G**,**H**) NiV-infected Vero cells with overexpression of METTL3, FTO, or ALKBH5, or knockdown of FTO or ALKBH5, were analyzed 48 h post-infection for NiV RNA levels by qRT-PCR. (**A**,**B**,**H**) Data are mean ± SEM (*n* = 3); ** *p* < 0.01, ns: not significant, unpaired Student’s *t*-test. (**D**,**G**) Data are mean ± SEM (*n* = 3); ** *p* < 0.01, ns: not significant, one-way ANOVA with Dunnett’s multiple comparisons test. (**C**) Western blot detection of the expression of ALKBH5 in Vero cells treated with siALKBH5. (**E**,**F**) Western blot analysis of FTO expression following FTO knockdown or overexpression.

The influence of m6A on NiV replication was examined by overexpressing METTL3 in Vero cells, which resulted in an increase in NiV RNA levels (Figure 4A). In contrast, ALKBH5 overexpression dramatically reduced viral replication (Figure 4B), while ALKBH5 knockdown enhanced NiV replication (Figure 4C,D). Moreover, neither overexpression nor knockdown of FTO significantly affected NiV replication (Figure 4E–H). These results confirm the critical role of ALKBH5 in regulating NiV methylation and demonstrate that m6A positively modulates NiV replication.

### 3.4. STM2457 as a Potential Antiviral Drug Against NiV

**Figure 5:**
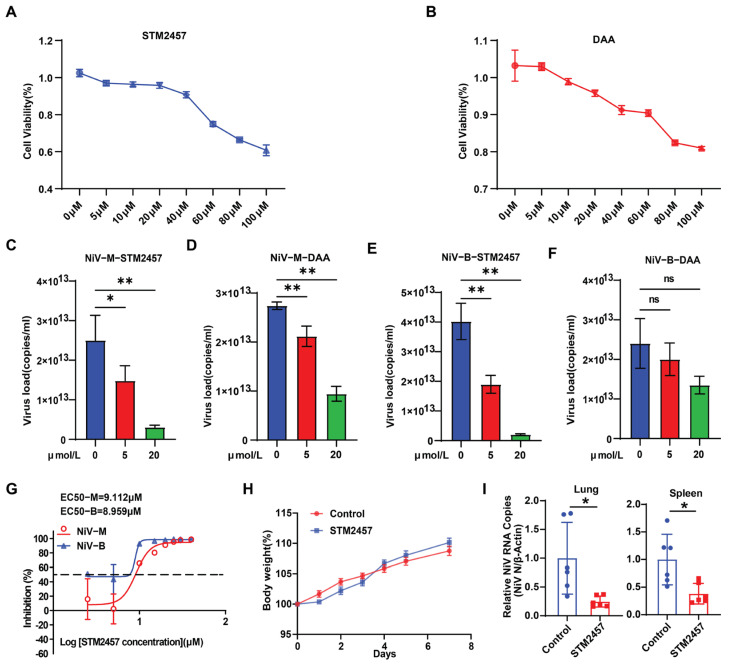
STM2457 as a potential antiviral drug against NiV. (**A**,**B**) CCK8 assay to evaluate the effect of different concentrations of STM2457 and DAA on cell viability. (**C**–**F**) Vero cells treated with STM2457 or DAA, infected with NiV-M or -B, were collected 48 h later, and NiV RNA copy number in the supernatant was quantified by qPCR. Data are mean ± SEM (*n* = 3); ** *p* < 0.01, * *p* < 0.05, ns, no significance (*p* > 0.05), one-way ANOVA with Dunnett’s multiple comparisons test. (**G**) Dose–response analysis of STM2457 in Vero cells infected with NiV-M and NiV-B; EC_50_ values were determined. (**H**) Hamster were administered 20 mg/kg STM2457 via intraperitoneal injection every other day, and body weight was monitored daily. (**I**) qPCR quantification of NiV RNA copies in lung and spleen tissues of hamsters following STM2457 treatment. Data are presented as mean ± SEM (*n* = 6); * *p* < 0.05, unpaired Student’s *t*-test with Welch’s correction.

Currently, no effective antiviral drugs are available for NiV. The potential of METTL3 inhibitor STM2457 and global methylation inhibitor DAA as antiviral agents was evaluated. Both compounds maintained cell viability above 95% at concentrations ≤20 μM, as determined by CCK8 assays (Figure 5A,B). Vero cells were subsequently treated with 0, 5, and 20 μM of STM2457 or DAA, followed by NiV infection and absolute quantification qPCR. Both inhibitors reduced NiV-M RNA copy numbers, with STM2457 demonstrating greater efficacy (Figure 5C,D). For NiV-B, STM2457 retained significant inhibitory activity (Figure 5E), whereas DAA showed minimal effect (Figure 5F). EC_50_ assays further confirmed the potent antiviral activity of STM2457, revealing a higher EC_50_ against NiV-M compared to NiV-B, which indirectly suggests a higher virulence of the NiV-M strain (Figure 5G).

We subsequently evaluated the effects of STM2457 on NiV replication in a hamster infection model. Initial experiments involved intraperitoneal administration of 20 mg/kg STM2457 every other day. Daily monitoring revealed consistent weight gain (Figure 5H), normal behavioral patterns (including food intake and urination), and healthy glossy fur without observable toxicity throughout the observation period. Based on these safety parameters, this concentration was used for subsequent antiviral efficacy testing. Three hours post-administration, animals were challenged with NiV via intraperitoneal injection, followed by an additional dose at 48 h intervals. Tissue analysis on day 4 post-infection demonstrated that STM2457 treatment significantly reduced NiV RNA copy numbers in both lung and spleen tissues compared to controls (Figure 5I). These findings collectively indicate that STM2457 effectively inhibits NiV replication in both cellular and animal models.

## 4. Discussion

This study investigated the interplay between m6A and NiV replication across two distinct strains (NiV-M and NiV-B). We mapped the m6A landscape of NiV genomic RNA and polyadenylated mRNAs, identified METTL3 and ALKBH5 as key regulators of viral replication, and demonstrated the methylation inhibitors shows potent anti-NiV activity. Our findings enhance the understanding of m6A dynamics in NiV infection and highlight a promising host-targeted antiviral strategy.

The functional significance of m6A modifications is closely tied to their location. In host mRNAs, m6A is predominantly enriched in 3′ untranslated regions (3′UTRs), near stop codons, and within long exons, while being relatively scarce in 5′UTRs, which promotes mRNA stability, export, and translation. Viral RNAs, however, exhibit divergent m6A distribution patterns across species, with modifications observed in 3′UTRs, 5′UTRs, and coding regions, regulating infection through distinct mechanisms. Notably, NiV exhibits a strain-dependent m6A landscape linked to replication efficiency. In its negative-sense genomic RNA, m6A clusters near the 3′ terminus—likely a conserved feature given that the 3′ end serves as the replication initiation site for RdRp. This enrichment may affect RdRp binding or template stability, similar to 3′ modifications in coronaviruses that enhance replication fidelity. Furthermore, NiV polyadenylated mRNAs show widespread m6A distribution across coding regions, akin to EV71 and SARS-CoV-2. While SARS-CoV-2 uses m6A to evade immune detection and EV71 relies on it for IRES-mediated translation, NiV’s segmented transcription strategy suggests even more complex regulatory roles. The higher m6A density in NiV-M compared to NiV-B may lead to enhanced translation or altered host factor recruitment, potentially explaining its superior replication. Future studies should explore how these modifications impact transcript stability, translation efficiency, and immune evasion in NiV.

NiV infection downregulates METTL3 and ALKBH5 while upregulating YTHDF readers in Vero cells, aligning with broader viral strategies to manipulate m6A dynamics. For instance, EV71 enhances METTL3 and YTHDF while suppressing FTO to promote replication. RSV and VSV have all been shown to enhance METTL3 expression [62,63,77,78]. Meanwhile, SARS-CoV-2 increased METTL3 levels in Vero E6 cells but suppressed METTL3 transcripts in lung samples from infected patients [50,51]. In contrast, infection with Japanese encephalitis virus (JEV) inhibited METTL3 expression in mouse brain tissue [79]. Despite global METTL3 downregulation, NiV sustains high viral RNA m6A levels, suggesting a potential ‘selective hijacking’ mechanism in which residual METTL3 might localize to replication complexes, ALKBH5 suppression could limit demethylation, and upregulated YTHDFs may enhance viral transcript stability. Cross-modification comparisons further underscore divergent strategies: while HCV exploits NSUN2-mediated m5C for RNA stability [80], SARS-CoV-2 repurposes NSUN2 to restrict replication [81]; EV71 leverages NAT10-dependent ac4C to boost IRES translation [82], whereas Kaposi’s sarcoma-associated herpesvirus (KSHV) co-opts ac4C-modified lncRNAs for immune evasion [83]. NiV epitomizes a “global suppression–local enrichment” strategy, refining host–virus conflicts through selective m6A machinery engagement, with broader implications for targeting RNA modifications in antiviral development.

To date, no clinically approved drugs exist for NiV, with current candidates—neutralizing antibodies (e.g., NIV41, m102.4) and polymerase inhibitors (e.g., remdesivir)—limited by epitope variability, rapid resistance, and insufficient blood–brain barrier penetration. Emerging host-directed strategies targeting RNA m6A modification offer a promising alternative: the METTL3 inhibitor STM2457 demonstrates robust efficacy against both NiV strains (NiV-M and NiV-B) by disrupting viral RNA methylation, while DAA, a broad methyltransferase inhibitor, further suppresses replication through similar mechanisms despite potential off-target effects. These m6A-targeting agents align with successful precedents: METTL3 inhibition curtails HSV-1 replication [71], while DZNep blocks SARS-CoV-2 transcription [84], underscoring the versatility of epitranscriptomic interventions. By leveraging conserved host machinery, m6A inhibitors reduce resistance risks and exhibit broad-spectrum potential across paramyxoviruses. Future efforts should prioritize optimizing CNS delivery (e.g., ferritin nanoparticle carriers for brain targeting) and integrating m6A modulators with neurotropic therapies like nanobody N425. This focus on RNA modification not only addresses NiV’s unique challenges but also highlights the broader promise of epitranscriptomic engineering in combating neurovirulent and emerging RNA viruses.

Our findings in Vero cells and hamsters contrast with prior reports in ferrets and primates, where NiV-B demonstrated superior replication capacity [17,85]. This discrepancy likely reflects host-specific factors, including interferon competence, tissue tropism, and immune microenvironments. Vero cells lack a functional interferon system, which may disproportionately enhance NiV-M replication. Hamsters, while immunocompetent, may mount a weaker respiratory inflammatory response, favoring the neurotropic adaptation of NiV-M. In contrast, ferrets and primates exhibit stronger respiratory inflammation and antiviral signaling, conditions more conducive to NiV-B replication. Since immune activation and inflammation are key contributors to NiV disease progression, these host-specific variations may explain the observed strain-dependent replication patterns. Our findings underscore the importance of using diverse experimental models to comprehensively assess NiV pathogenesis.

By linking m6A modification to NiV strain pathogenicity, this work redefines epitranscriptomic regulation as a critical virulence factor in paramyxoviruses. Translationally, METTL3/ALKBH5 modulators could serve as first-line therapeutics during NiV outbreaks, while strain-specific m6A signatures may inform rapid virulence diagnostics. Future efforts should prioritize, such as single-nucleotide resolution mapping to identify m6A “hotspots” regulating NiV polymerase processivity or nucleocapsid assembly. Artificial intelligence-driven drug optimization: Structure prediction tools like AlphaFold [86] could model m6A enzyme–viral RNA interfaces for inhibitor design, while organoid-based platforms enable high-throughput screening of m6A-targeted libraries. Cross-species m6A dynamics: Profiling m6A landscapes in NiV-infected bat, pig, and human cells may reveal epigenetic “adaptation signatures” driving zoonotic transmission.

## Figures and Tables

**Table 1:** Analysis of the number of AAC and GAC motifs in the genomes and mRNAs of NiV-M and NiV-B.

m6A Motif	Strain Type	Forward Strand	Reverse Strand	All
AAC	NiV-M	331	258	589
	NiV-B	328	255	583
GAC	NiV-M	244	174	418
	NiV-B	238	181	419

**Table 2:** Assessment of changes in m6A-related proteins following NiV infection based on transcriptomic analysis data.

	log2 (NIV-M/Mock)	*p* Value	log2 (NIV-B/Mock)	*p* Value
METTL3	−0.61747	0.59795	−0.88308	0.45441
METTL14	−0.37466	5.55 × 10^−6^	−0.39354	3.26 × 10^−6^
ALKBH5	−0.66281	0.51862	0.34017	0.68319
YTHDF1	0.50838	5.1 × 10^−13^	0.55473	9.4 × 10^−14^
YTHDF2	0.80903	0.18382	0.40173	0.50732
YTHDF3	0.22008	0.74839	0.05200	0.93705

## Data Availability

The data that support the findings of this study are available from the corresponding author, H.H., upon reasonable request.
